# Discrete gait characteristics are associated with m.3243A>G and m.8344A>G variants of mitochondrial disease and its pathological consequences

**DOI:** 10.1007/s00415-013-7129-2

**Published:** 2013-10-23

**Authors:** Brook Galna, Jane Newman, Djordje G. Jakovljevic, Matthew G. Bates, Andrew M. Schaefer, Robert McFarland, Douglass M. Turnbull, Michael I. Trenell, Gráinne S. Gorman, Lynn Rochester

**Affiliations:** 1 Clinical Ageing Research Unit, Campus for Ageing and Vitality, Institute for Ageing and Health, Newcastle University, Newcastle Upon Tyne, UK; 2 Wellcome Trust Centre for Mitochondrial Research and NIHR Biomedical Research Centre for Ageing and Age-related Disease, Newcastle University, Newcastle upon Tyne, UK; 3 MoveLab, The Medical School, Newcastle University, 4th Floor William Leech Building, Newcastle upon Tyne, UK

**Keywords:** Mitochondrial disease, Gait, Disease severity, Genotype, Cerebellum

## Abstract

Mitochondrial disease is complex and variable, making diagnosis and management challenging. The situation is complicated by lack of sensitive outcomes of disease severity, progression, contributing pathology and clinical efficacy. Gait is emerging as a sensitive marker of pathology; however, to date, no studies have quantified gait in mitochondrial disease. In this cross-sectional study, we quantified gait characteristics in 24 patients with genetically confirmed mitochondrial disease (m.3243A>G and m.8344A>G) and 24 controls. Gait was measured using an instrumented walkway according to a predefined model with five domains hypothesised to reflect independent features of the neural control of gait in mitochondrial disease, including: pace (step velocity and step length); rhythm (step time); variability (step length and step time variability); asymmetry (step time asymmetry); and postural stability (step width, step width variability and step length asymmetry). Gait characteristics were compared with respect to controls and genotype. Additional measures of disease severity, pathophysiology and imaging were also compared to gait to verify the validity of gait characteristics. Discrete gait characteristics differed between controls and mitochondrial disease groups, even in relatively mildly affected patients harbouring the m.3243A>G mutation. The pattern of gait impairment (increased variability and reduced postural control) was supported by significant associations with measures of disease severity, progression, pathophysiology and radiological evidence of cerebellar atrophy. Discrete gait characteristics may help describe functional deficits in mitochondrial disease, enhance measures of disease severity and pathology, and could be used to document treatment effects of novel therapies.

## Introduction

Mitochondrial disorders are one of the commonest inherited neuromuscular disorders and present with a highly variable and complex pattern of neurological and systemic involvement [1]. Whilst progress has been made identifying definitive genotypes in mitochondrial diseases, this is of limited help to the clinician or patient because of considerable heterogeneity in phenotype, which can be only partly explained by the level of mutant and wild type mtDNA present (heteroplasmy). Phenotypic heterogeneity is reflected by the highly variable presentation of clinical features [2]. The relationship between clinical outcomes, genotype and heteroplasmy is therefore complex and current clinical outcomes do not accurately discriminate for genotype or heteroplasmy, thereby limiting understanding of disease evolvement, underlying pathology and appropriate interventions. A recent Cochrane review echoes this sentiment concluding that there was no clear evidence to support the use of any intervention in mitochondrial disorders with heterogeneity in outcome measures limiting comparison between trials [3].

Gait is emerging as a powerful measurement tool to identify markers of incipient pathology, inform diagnostic algorithms and disease progression, and measure efficacy of interventions [4]. Discrete features of gait (such as variability in the magnitude of stride to stride fluctuations) are highly sensitive to the effects of ageing and pathology [5–7], including incipient pathology associated with increased genetic risk factors, making gait a potentially useful metric to aid the neurologist [8, 9]. To date however no studies have carried out a detailed quantification of gait in patients with mitochondrial disease.

We therefore aimed to (1) describe the pattern of gait impairments in patients with different genetic variants of mitochondrial disease; and (2) explore the relationships between gait characteristics with measures of mitochondrial disease severity, its underlying pathophysiological consequences (such as myopathy and cerebellar atrophy) and clinical and pathological measures. We, firstly, hypothesised that gait characteristics would differ between mitochondrial disease and control groups. Secondly, we hypothesised that the pattern of deficit in gait would differ with respect to genotype. Thirdly, gait characteristics would be selectively associated with underlying markers of disease (heteroplasmy) and its pathological consequences (e.g., muscle weakness and ataxia). Lastly, we dichotomised a subgroup of the cohort for presence (or absence) of cerebellar atrophy as shown on MR and compared the gait characteristics of both groups to verify our findings.

## Subjects and methods

### Subjects and study design

Patients with mitochondrial disease due to either the m.3243A>G (18 patients) or 8344A>G mutations were recruited from a mitochondrial outpatient clinic as part of an exercise intervention study and had to be able to perform the walking assessment safely. We aimed to recruit 12 people each in the two arms of the intervention. Sample size estimates for the exercise study were based on a change in peak oxygen consumption following a similar exercise intervention, allowing for 25 % attrition [10]. A convenience sample of age-matched and sex-matched control participants was also recruited. Ethical approval was provided by the Sunderland Research Ethics Committee, United Kingdom, and written informed consent was obtained from participants prior to testing.

### Demographic, clinical and pathological measures

Age, sex, and height were recorded for all participants. Disease severity was assessed using the Newcastle Mitochondrial Disease Adult Scale (NMDAS) [11], a validated measure of disease burden and surrogate for phenotypic severity, prospectively at the time of gait measurement. Mitochondrial mutation load was assessed using urinary epithelial cells [12]. Physiological markers of mitochondrial disease were collected as part of a larger exercise study (manuscript in preparation) as follows: (1) energy expenditure was assessed using a body-worn multi-sensor array (SenseWear Pro3, Bodymedia Inc, Pennsylvania, USA 2.5), which was worn by the participant for seven days in the community; (2) exercise capacity (peak oxygen consumption) was evaluated using cardiopulmonary exercise testing, which was performed as previously reported [13] (During all visits, a stepped incremental workload test (~10–20 W/min) was conducted to elicit a symptom-limited maximum oxygen uptake and heart rate response); (3) muscle strength testing evaluated hip flexor and extensor strength and was performed by the same investigator (JN) using an isokinetic dynamometer (CSMI HUMAC^®^/NORM testing and rehabilitation system) at a speed of 60°/s [ 14]. (Participants underwent familiarization with six sub-maximal contractions and then performed six maximal repetitions with consistent verbal encouragement); and (4) where available, brain MRI images were reviewed (GSG) for the presence or absence of global cerebellar atrophy retrospectively. GSG was blinded to the gait results but not genotype at the time of assessment.

### Quantitative gait assessment

Gait was assessed within a week of the clinical and pathological measures in a gait laboratory setting. Participants performed a block of three 12 m walks at their “comfortable walking pace.” Gait was assessed using a 7 m long × 0.6 m wide instrumented mat (Platinum model Gaitrite, software version 4.5, CIR systems, USA). The mat was placed in the centre of the walkway to ensure participants were walking at a steady speed whilst gait was measured. Previous studies have verified the Gaitrite mat to be a valid and reliable method for measuring gait [15]. Description of the method of calculating gait characteristics is reported in detail elsewhere [16]. Gait variability was calculated from standard deviation of left and right steps, which were then combined, and gait asymmetry was calculated as the absolute difference between left and right steps.

### Data processing and analysis

We quantified gait according to a predefined model with five domains hypothesised to reflect independent features of the neural control of gait, which provided a theoretical framework to describe the features of gait associated with mitochondrial disease and its genotypes [17] and included: pace (step velocity and step length); rhythm (step time); variability (step length and step time variability); asymmetry (step time asymmetry); and postural stability (step width, step width variability and step length asymmetry). Data for individual steps for each condition were extracted from the Gaitrite database using Microsoft Access 2007.

### Statistics

Non-parametric Kruskal-Wallace tests were used to test for group differences in gait characteristics. Post-hoc Mann–Whitney *U* tests were considered significant if *p* < 0.05 after Holm–Bonferroni corrections. Associations between gait and disease severity, mutation load, and pathophysiological variables were assessed using Spearman Rho correlations. To avoid misleading findings, we limited our correlation analysis between gait and the NMDAS to the total NMDAS and exercise tolerance, gait stability, cerebellar ataxia, and myopathy subscales. To accommodate for multiple correlations, only correlations with a *p* < 0.01 were considered statistically significant.

## Results

Sixty people who attended a specialist outpatient clinic were invited to participate in an exercise study for mitochondrial disease. Of these, four were ineligible because they were unable to undergo an MRI, and one person had a recent fracture. Eleven did not wish to participate because of time commitments or ill health, and 21 failed to reply. All 24 who consented went on to be tested. Of the 24 patients with mitochondrial disease, 18 patients presented with the m.3243A>G mutation and six patients with the m.8344A>G mutation. We also tested 24 age-matched and sex-matched controls. Participant demographic and clinical descriptors are presented in Table 1. Both the score on the NMDAS (*p* = 0.023) and urinary mutation load (*p* = 0.001) was significantly lower for m.3243A>G than for m.8344A>G. People with mitochondria disease also demonstrated reduced community energy expenditure (*p* = 0.001), exercise capacity (*p* = 0.001) and hip flexion (*p* = 0.002) and extension (*p* = 0.003) strength compared to controls.

**Table 1:** Individual and group median (quartiles) demographic characteristics of patients with mitochondrial disease and control subjects

Group	Age	Sex	Height (cm)	Body mass (kg)	BMI (kg m^−2^)	Mutation load (%)	NMDAS	Phenotype
m.3243A>G	50	f	169	63.4	22.2	34	3	Migraine, fatigue, hypothyroidism, myalgia, constipation, hypertension, dyslipidaemia, coeliac disease
	58	f	173	52.1	17.4	59	12	Hearing loss, ataxia, constipation, underweight, myopathy, myalgia, exercise intolerance
	58	m	186	89.8	25.9	66	30	Hearing loss, diabetes, ataxia, retinopathy, constipation, hypertension, dysarthria, myopathy, neuropathy, exercise intolerance
	39	m	172	74.6	25.2	80	17	Hearing loss, diabetes, migraine, fatigue, hypothyroidism, ataxia, constipation, exercise intolerance
	37	f	164	50.7	18.9	48	10	Hearing loss, diabetes, exercise intolerance, ataxia, dysarthria, asthma
	42	m	176	83.7	27.0	82	12	Hearing loss, diabetes, migraine, ataxia, depression, exercise intolerance
	42	f	154	49.0	20.7	43	15	Hearing loss, diabetes, ataxia, constipation, depression, exercise intolerance, short stature
	47	m	182	64.0	19.3	63	28	Hearing loss, diabetes, fatigue, ataxia, myalgia, depression, myopathy, neuropathy, ptosis, PEO
	38	f	164	54.2	20.1	53	11	Hearing loss, migraine, constipation, myopathy, fatigue, exercise intolerance, asthma
	22	m	183	59.2	17.7	89	17	Hearing loss, migraine, epilepsy, ataxia, constipation, underweight, exercise intolerance, fatigue
	53	f	164	59.1	22.0	22	10	Hearing loss, ataxia, retinopathy, constipation, myopathy, exercise intolerance
	25	m	174	49.8	16.5	90	26	Hearing loss, migraine, ataxia, retinopathy, constipation, depression, dysarthria, myopathy, exercise intolerance, short stature, asthma
	18	f	154	39.5	16.7	59	9	Hearing loss, retinopathy, underweight, myopathy, exercise intolerance, short stature
	24	f	155	64.0	26.7	72	27	Hearing loss, migraine, ataxia, retinopathy, constipation, depression, dysarthria, myopathy, exercise intolerance, short stature
	55	m	179	104.5	32.6	76	14	Hearing loss, diabetes, depression
	36	m	179	72.4	22.6	80	4	Migraine, exercise intolerance
	50	m	162	64.6	24.6	87	23	Hearing loss, exercise intolerance, ataxia, myopathy, fatigue, depression, retinopathy, epilepsy, encephalopathy, cognitive decline, stroke-like episodes
	55	f	153	45.8	19.5	68	25	Hearing loss, diabetes, myopathy, exercise intolerance, ataxia, constipation, depression, retinopathy, PEO, ptosis, short stature, mild dysphagia, hypertension
m.8344A>G	46	m	163	70.9	26.7	77	19	Mild concentric LVH, lipomata, exercise intolerance, dysphagia, myoclonic jerks, ataxia, seizures, neuropathy, fasciculations, constipation, hearing loss, myopathy, parenchymal lung disease
	28	m	173	51.7	17.3	95	55	Hearing loss, fatigue, epilepsy, ataxia, retinopathy, constipation, dysarthria, myopathy, myoclonus, neuropathy, exercise intolerance, underweight
	59	f	162	69.8	26.6	75	19	Myoclonus, deaf, diabetes, lipomata, myopathy, mild concentric LVH
	25	m	176	68.0	22.0	94	48	Epilepsy, ataxia, retinopathy, depression, dysarthria, exercise intolerance, myopathy, myoclonus
	28	m	180	56.2	17.3	93	35	Hearing loss, migraine, epilepsy, ataxia, constipation, underweight, depression, dysarthria, myopathy, neuropathy, exercise intolerance
	38	m	160	76.5	29.9	94	58	Lipomata, hearing loss, migraine, epilepsy, ataxia, retinopathy, depression, dysarthria, myopathy, myoclonus, neuropathy, exercise intolerance, short stature
m.3243A>G	42 (36,52)	f9 m9	170 (162, 178)	61.3 (51.0, 70.4)	21.3 (18.9, 25.1)	67.0 (51.8, 80.5)	15 (10, 25)	
m.8344A>G	33 (28,44)	f1 m5	168 (162, 175)	68.9 (59.1, 70.6)	24.3 (18.5, 26.7)	93.5 (76.5, 94.3)	42 (19, 56)	
Control	41 (32,51)	f10 m14	170 (163, 178)	75.4 (69.7, 84.4)	25.2 (24.1,28.9)	–	–	

NMDAS, Newcastle Mitochondrial Disease Adult Scale, consists of 29 subscales scored from 0 to 5, with a higher score indicating more severe symptoms

### Gait deficits associated with mitochondrial disease

**Fig. 1:**
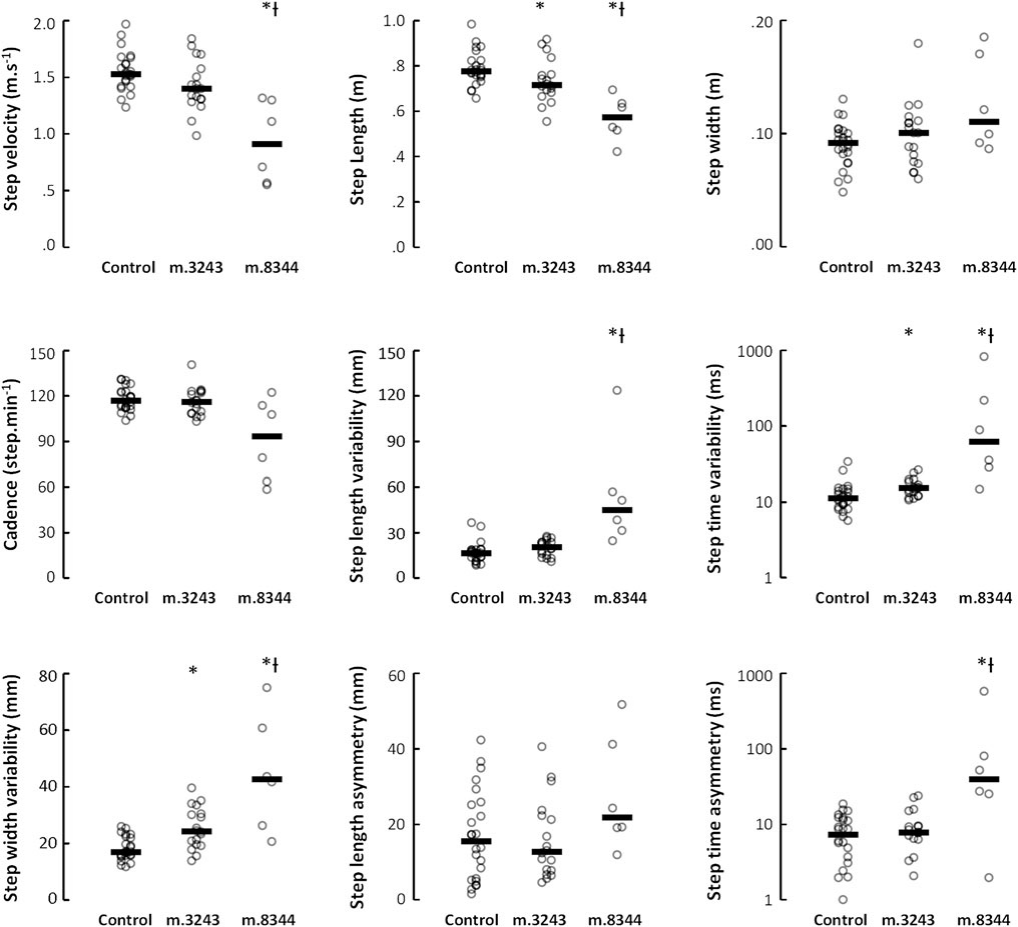
Individual and group gait performance. The *solid bar* represents the median value with individual scores displayed around the median. *Indicates a significant difference compared to control participants. ^†^Indicates a significant difference between m.3243 and m.8344 genotypes. A *p* value of ≤0.05 after Holm–Bonferroni corrections was considered significant

Compared to controls, patients with mitochondrial disease had significantly reduced gait speed, step length and increased variability in step time, step length and step width (Table 2; Fig. 1). When described by genotype; however, a more selective picture emerged. Patients with the m.8344A>G mutation were globally impaired for all gait characteristics except cadence, step width and step length asymmetry compared to both the control and m.3242A>G group (Table 2; Fig. 1). Patients with the m.3243A>G mutation had a significantly shorter step length and increased variability in step time and step width compared to controls.

**Table 2:** Group median (quartiles) of gait performance, and *p* values Kruskal–Wallis and Mann–Whitney *U* tests for group differences in gait performance

Gait domain	Gait variables	Controls	m.3243A>G	m.8344A>G	Mann whitney *U* tests *(p*)				
					Kruskal–Wallis (*p*)	Controls and mtDNA disease	Controls and m.3243A>G	Controls and m.8344	m.3243A>G and m.8344A>G
Pace	Step velocity (m.s^−1^)	1.52 (1.43, 1.66)	1.40 (1.30, 1.60)	0.91 (0.56, 1.30)	*0.002*	*0.002*	0.042	*<0.001*	*0.003*
	Step length (m)	0.78 (0.75, 0.82)	0.71 (0.68, 0.78)	0.57 (0.49, 0.65)	*0.001*	*0.001*	*0.024*	*<0.001*	*0.001*
Rhythm	Cadence (steps min^−1^)	117 (112, 123)	116 (108, 123)	93 (62, 116)	0.*046*	0.155	0.525	0.021	0.040
Asymmetry	Step time asymmetry (m.s)	7.3 (2.6, 11.9)	7.8 (3.5, 18.4)	39.4 (19.2, 202.6)	*0.035*	0.149	0.629	*0.006*	*0.009*
Variability	Step length variability (mm)	16.3 (13.5, 18.5)	20.3 (14.5, 23.9)	44.8 (29.6, 73.4)	*<0.001*	*0.004*	0.067	*<0.001*	*<0.001*
	Step time variability (ms)	11.1 (8.6, 14.6)	15.2 (11.9, 18.5)	61.5 (24.8, 368.1)	*0.005*	*<0.001*	*0.004*	*<0.001*	*0.002*
Postural control	Step width (m)	0.092 (0.076, 0.102)	0.100 (0.075, 0.112)	0.110 (0.090, 0.174)	0.220	0.112	0.297	0.065	0.251
	Step width variability (mm)	16.9 (15.6, 22.3)	24.2 (19.4, 31.2)	42.6 (24.9, 64.3)	*0.016*	*<0.001*	*0.001*	*<0.001*	*0.022*
	Step length asymmetry (mm)	15.5 (5.3, 25.6)	12.7 (7.4, 22.6)	21.7 (17.3, 43.8)	*0.019*	0.536	0.980	0.116	0.056

Raw *p* values are presented. Significant group differences after correcting for Holm–Bonferroni corrections are highlighted in italics

### Relationship of gait and clinical burden of disease

Higher clinical disease burden (NMDAS) correlated with reduced walking speed, step length, and increased step width variability and step length asymmetry. The pattern of association was different for the individual subscales with additional features emerging in respect to variability and asymmetry (Table 3).

**Table 3:** Correlation between gait performance, total NMDAS, and NMDAS subscales

Gait domain	Gait variables	NMDAS	NMDAS subscale			
			Exercise tolerance	Gait stability	Myopathy	Cerebellar ataxia
Pace	Step velocity	−*0.632* (*0.001*)	*−0.733* (*0.001*)	*−0.569* (*0.005*)	*−*0.393 (0.064)	*−*0.425 (0.043)
	Step length	*−0.567* (*0.004*)	*−0.744* (*0.001*)	*−0.599* (*0.003*)	*−*0.472 (0.023)	*−*0.377 (0.076)
Rhythm	Cadence	*−*0.409 (0.047)	*−0.525* (*0.010*)	*−*0.417 (0.048)	*−*0.074 (0.736)	*−*0.311 (0.149)
Asymmetry	Step time asymmetry	0.449 (0.028)	*0.622* (*0.002*)	0.456 (0.029)	*0.632* (*0.001*)	0.454 (0.030)
Variability	Step length variability	0.353 (0.090)	0.501 (0.015)	0.463 (0.026)	0.214 (0.326)	*0.545* (*0.007*)
	Step time variability	0.161 (0.452)	0.412 (0.051)	0.347 (0.105)	0.067 (0.762)	*0.531* (*0.009*)
Postural control	Step width	0.394 (0.057)	0.424 (0.044)	0.438 (0.036)	0.357 (0.094)	0.453 (0.030)
	Step width variability	*0.616* (*0.001*)	0.320 (0.137)	*0.613* (*0.002*)	0.339 (0.113)	*0.635* (*0.001*)
	Step length asymmetry	*0.623* (0*.001*)	0.290 (0.179)	0.286 (0.186)	*0.540* (*0.008*)	0.429 (0.041)

NMDAS Newcastle Mitochondrial Disease Adult Scale, subscales range from 0 to 5, with a higher score indicating more severe symptoms. Significant correlations presented in italics (*p* ≤ 0.01, two-tailed)

### Relationship of gait with pathophysiological burden of disease

We explored gait characteristics with respect to a battery of pathophysiological markers of mitochondrial disease (mutation load, energy expenditure, exercise capacity [peak oxygen consumption], and muscle strength) (Table 4). For this analysis, we concentrated on the m.3242A>G group because we were interested in those characteristics that were sensitive to early subtle impact of disease independent of the compensatory changes observed in the m.8344A>G group. Increased mutation load was significantly associated with increased step time asymmetry and step width variability (Table 4). The relationship between gait and energy expenditure, peak exercise capacity and muscle strength was less clear. There were moderate correlations between reduced step length and velocity (consistent with an independent domain of gait representing pace); and impaired muscle strength (hip flexors and extensors), peak exercise capacity and energy expenditure; however, several of these did not reach statistical significance. No other variables were related to the physiological characteristics.

**Table 4:** Spearman Rho correlations between mutation load, energy expenditure, peak exercise capacity and muscle strength variables and gait outcomes in people with Mitochondrial disorder

Gait domain	Gait variables	Mutation load (%)	Community energy expenditure (cals)	Exercise capacity (peak oxygen consumption) (ml kg min)	Hip flexor strength (nm/kg)	Hip extensor strength (nm/kg)
Pace	Step velocity	−0.294 (0.164)	0.305 (0.157)	0.475 (0.019)	0.441 (0.045)	0.453 (0.039)
	Step length	−0.228 (0.285)	0.433 (0.039)	*0.605* (*0.002*)	0.513 (0.017)	0.506 (0.019)
Rhythm	Cadence	−0.406 (0.049)	−0.023 (0.918)	0.178 (0.405)	0.067 (0.773)	0.192 (0.404)
Asymmetry	Step time asymmetry	*0.566* (*0.004*)	−0.093 (0.673)	−0.336 (0.109)	−0.343 (0.128)	−0.457 (0.037)
Variability	Step length variability	0.383 (0.064)	−0.186 (0.396)	−0.209 (0.328)	−0.182 (0.430)	−0.173 (0.454)
	Step time variability	0.154 (0.474)	−0.256 (0.239)	−0.154 (0.473)	−0.318 (0.159)	−0.203 (0.378)
Postural control	Step width	0.090 (0.674)	0.201 (0.359)	−0.003 (0.990)	−0.069 (0.767)	−0.021 (0.929)
	Step width variability	*0.605* (*0.002*)	0.069 (0.754)	−0.165 (0.440)	−0.333 (0.140)	−0.288 (0.205)
	Step length asymmetry	0.392 (0.058)	0.078 (0.723)	−0.319 (0.129)	−0.181 (0.431)	−0.206 (0.369)

Significant correlations presented in italics (*p* < 0.05, two-tailed)

Structural magnetic resonance brain images were available for 13 of the 24 mitochondrial participants. Because there were fewer participants with available imaging, we pooled both genotypes to explore the relationship of cerebellar atrophy as a marker of ataxia and gait characteristics. Cerebellar atrophy was defined if the cerebellum was small with shrunken folia and large cerebellar fissures. Of those subjects four were the m.8344A>G genotype and all had cerebellar atrophy while nine represented the m.3243A>G genotype and of those six had evidence of atrophy. Participants with cerebellar atrophy walked with significantly greater step width (*p* = 0.049), step width variability (*p* = 0.049), and step length asymmetry (*p* = 0.014), consistent with domains of gait that represent postural control and variability.

## Discussion

To our knowledge, this is the first study to quantify gait in genetically confirmed mitochondrial disease. We used a robust model to capture the complexity of gait, which enabled us to differentiate mitochondrial genotypes and identify the pattern of gait deficits potentially reflecting differences in the underlying pathophysiology and neural control of locomotion. Our findings confirm our hypotheses and show that gait differed in a sensitive and selective manner with respect to mitochondrial disease and genotype even in relatively mildly affected patients with m.3243A>G. Associations with clinical disease burden as assessed by NMDAS and pathophysiological markers support the use of gait as a robust clinical measure of disease severity.

### Gait deficits associated with mitochondrial disease and genotype

Gait impairment was observed in both the mitochondrial disease group and individual genotypes compared to controls. The m.8344A>G genotype was globally impaired in all gait characteristics except cadence, step width and step length asymmetry which is possibly not surprising given the very marked impairment of physical function and high level of clinical symptoms present in this group. In contrast, fewer gait characteristics differed between the m.3243A>G genotype and controls reflecting their higher level of performance whilst highlighting difficulties maintaining adequate pace (step length), low magnitude of variability (step time variability) and postural control (step width variability) when walking [16]. These results also suggest that different pathological processes may underpin gait impairments such that even patients with the least severe m.3243A>G genotype could be differentiated on the basis of their gait characteristics.

### Does gait reflect the burden of disease?

We explored the associations between gait and clinical disease severity (NMDAS) for the total mitochondrial disease group (m.8344A>G and m.3243A>G). Patients with more severe symptoms (NMDAS) had greater gait impairment. Some of the gait characteristics that differed between people with mitochondrial disease and controls were different from those associated with the NMDAS suggesting caution in selection of outcomes, if the purpose of gait is to provide a correlate of disease severity. Gait speed, step length, and step width variability, however, were both sensitive to group differences and are associated with the NMDAS, guiding variable selection. Associations were observed for gait outcomes with exercise, gait and cerebellar sub-scales of the NMDAS, highlighting the underlying pathological contribution. The association between gait and the myopathy subscale was not so clear with gait asymmetry emerging as the only significant characteristic, which was surprising and difficult to explain. The six-item myopathy subscale features both upper and lower limb ratings, which may explain the poor association.

### Does gait reflect the pathological consequences of mitochondrial disease?

We were interested to explore the relationship between gait characteristics and the underlying pathophysiology (heteroplasmy), ataxia, muscle strength, and peak exercise capacity). The m.3243A>G genotype had high performing individuals (as determined by normal gait speed), and the gait impairments, therefore, most likely reflect the primary features of mitochondrial disease rather than compensatory changes related to reduced mobility. In contrast, the more global gait deficits in the m.8344A>G genotype reflect a combination of primary disease, deconditioning and compensation. We, therefore, focussed on the m.3243A>G genotype to explore the pathophysiological consequences of mitochondrial disease and used a model of gait as a theoretical framework to interpret the pattern of deficit with respect to their pathophysiological correlates.

Gait performance was significantly related to heteroplasmy for discrete characteristics (increased step width variability and step time asymmetry). Step width variability was also significantly greater in this group compared to controls. It is possible that these discrete features of gait reflect the underlying central pathological disease burden warranting further investigation.

Ataxia was also a common clinical symptom in this cohort. Cerebellar dysfunction is a major cause of gait ataxia and studies have shown that balance impairment and increased variability of global and segmental gait characteristics makes an important contribution [18–20]. Variability describes the magnitude of stride to stride fluctuations in gait allowing smooth, consistent rhythmical stepping movements. The cerebellum plays a role in control of timing and coordination of movement, making increased variability a likely consequence of cerebellar atrophy [21, 22]. Our findings support a role for cerebellar atrophy underpinning gait disturbance in mitochondrial disease. We found selective early changes in the generation of consistent steps leading to increased step time variability. In addition, increased step width variability reflects lateral instability and is implicated as a marker of impaired postural control consistent with our gait model and findings in older adults [17, 23]. Given the clinical presentation of these genotypes [2] in our group, it is highly likely that muscle pathology and ataxia either in isolation or in combination contribute in a selective manner to gait reflected in the subtle changes observed. This is also substantiated by significant correlations with cerebellar domains of the NMDAS, selective association with mutation load and evidence of increased cerebellar atrophy from a subset of participants for whom MR data was available.

Muscle strength is reflected in gait by step length, which determines the ability to maintain an adequate pace of locomotion. Reduced step length is, therefore, most likely a consequence of muscle weakness and myopathy. This is reinforced by the significant association of peak exercise capacity with step length. Together these features suggest an early and subtle contribution of muscle strength to generation of an adequate pace of locomotion as represented by step length and walking speed.

Although this study is the first to provide a description of gait impairment in patients with mitochondrial disease, we still do not know how quickly gait deteriorates with disease progression or how responsive gait impairments are to potential therapeutic intervention. In addition, we were only able to investigate gait impairment in two genotypes and in very small numbers and so our findings should be interpreted with this in mind and also may not extend to patients with other genotypes. However, given that ataxia is prevalent in our centre’s cohort of patients with mitochondrial disease with nearly one-third with documented ataxia (267/945 patients) and, less than half of these cases attributable to the genotypes under investigation here (71 patients harboring m.3243A>G mutation and 18 patients harboring m.8344A>G mutation), we postulate that our findings are transferrable to other genotypes, but further assessment is required. Seven patients from the total group had nerve conduction studies performed and out of these only three had electrophysiological evidence of a sensory-motor axonal neuropathy, and, although we concede that evidence is limited, it is unlikely that neuropathy is a significant contributor to the gait features identified. These findings also enhance opportunities for early targeted intervention at a time when there is a greater capacity to comply with therapy and avoid secondary consequences of disuse. In the absence of pharmacological evidence targeted physical therapy, it could be useful to address gait deficit early in the disease process when the deficits are relatively mild [24].

As identified in a recent Cochrane review [3], identification and harmonisation of sensitive outcomes is essential across centres and trials. Gait deficits are associated with mitochondrial disease and genotype in a selective fashion, which seems to reflect the clinical burden of disease and its pathological consequences. Furthermore, gait is easily quantifiable and sensitive to change in other clinical populations, therefore, quantification of discrete gait characteristics may enhance clinical measures of disease severity such as the NMDAS, pathology and efficacy of novel therapies. Although this was a small study, the results are interesting and warrant further longitudinal investigation as well as examination into the influence of gait deficits in mitochondrial disease on mobility and falls in the community.

## References

[1] McFarland R, Taylor R, Turnbull D (2010). A neurological perspective on mitochondrial disease. Lancet Neurol.

[2] Chinnery P, Howell N, Lightowlers R (1997). Molecular pathology of MELAS and MERRF. The relationship between mutation load and clinical phenotypes. Brain.

[3] Pfeffer G, Majamaa K, Turnbull D et al (2012) Treatment for mitochondrial disorders. Cochrane Database of Syst Rev 4:CD00426 10.1002/14651858.CD004426.pub3 PMC7201312 22513923

[4] Lord S, Galna B, Rochester L (2013) Moving forward on gait measurement: Towards a more refined approach. Mov Disord. doi:10.1002/mds.25545 10.1002/mds.25545 24132841

[5] Baltadjieva R, Giladi N, Gruendinger L (2006). Marked alterations in the gait timing and rhythmicity of patients with de novo Parkinson’s disease. Eur J Neurosci.

[6] Rochester L, Yarnall AJ, Baker MR (2012). Cholinergic dysfunction contributes to gait disturbance in early Parkinson’s disease. Brain.

[7] Verghese J, Wang C, Lipton RB (2007). Quantitative gait dysfunction and risk of cognitive decline and dementia. J Neurol Neurosurg Psychiatry.

[8] Mirelman A, Gurevich T, Giladi N (2011). Gait alterations in healthy carriers of the LRRK2 G2019S mutation. Ann Neurol.

[9] Rao AK, Muratori L, Louis ED (2008). Spectrum of gait impairments in presymptomatic and symptomatic Huntington’s disease. Mov Disord.

[10] Taivassalo T, Gardner JL, Taylor RW (2006). Endurance training and detraining in mitochondrial myopathies due to single large-scale mtDNA deletions. Brain.

[11] Schaefer AM, Phoenix C, Elson JL (2006). Mitochondrial disease in adults: a scale to monitor progression and treatment. Neurology.

[12] Whittaker RG, Blackwood JK, Alston CL (2009). Urine heteroplasmy is the best predictor of clinical outcome in the m.3243A>G mtDNA mutation. Neurology.

[13] Jakovljevic DG, Moore SA, Tan L-B (2012). Discrepancy between cardiac and physical functional reserves in stroke. Stroke.

[14] Dvir Z (2004). Isokinetics: muscle testing, interpretation, and clinical applications.

[15] Menz HB, Latt MD, Tiedemann A (2004). Reliability of the GAITRite^®^ walkway system for the quantification of temporo-spatial parameters of gait in young and older people. Gait Posture.

[16] Galna B, Lord S, Rochester L (2013). Is gait variability reliable in older adults and Parkinson’s disease? Towards an optimal testing protocol. Gait Posture.

[17] Lord S, Galna B, Verghese J et al (2012) Independent domains of gait in older adults and associated motor and non-motor attributes: validation of a factor analysis approach. J Gerontol Ser A Biol Sci Med Sci 68(7):820–827 10.1093/gerona/gls255 23250001

[18] Ilg W, Golla H, Thier P (2007). Specific influences of cerebellar dysfunctions on gait. Brain.

[19] Morton S, Bastian A (2004). Cerebellar control of balance and locomotion. Neurosci.

[20] Serrao M, Pierelli F, Ranavolo A et al (2012) Gait pattern in inherited cerebellar ataxia. Cerebellum 11(1):194–211 10.1007/s12311-011-0296-8 21717229

[21] Diedrichsen J, Criscimagna-Hemminger S, Shadmehr R (2007). Dissociating timing and coordination as functions of the cerebellum. J Neurosci.

[22] Spencer R, Zelaznik H, Diedrichsen J (2003). Disrupted timing of discontinuous but not continuous movements by cerebellar lesions. Science.

[23] Brach JS, Berlin JE, VanSwearingen JM (2005). Too much or too little step width variability is associated with a fall history in older persons who walk at or near normal gait speed. J Neuroeng Rehabil.

[24] Miyai I, Ito M, Hattori N (2012). Cerebellar ataxia rehabilitation trial in degenerative cerebellar diseases. Neurorehabil Neural Repair.

